# Electronic Properties and Stacking Ordering in Layered GeTe-Rich (GeTe)_*m*_(Sb_2_Te_3_)_*n*_

**DOI:** 10.1021/acsaelm.5c01185

**Published:** 2025-10-07

**Authors:** Flavia Righi Riva, Stefano Cecchi, Simone Prili, Omar Abou El Kheir, Ernesto Placidi, Marco Sbroscia, Adriano Diaz Fattorini, Sabrina Calvi, Massimo Longo, Marco Bernasconi, Raffaella Calarco, Fabrizio Arciprete

**Affiliations:** 1 Department of Physics, 9318 University of Rome Tor Vergata, Via della Ricerca Scientifica 1, Roma 00133, Italy; 2 Department of Materials Science, University of Milano-Bicocca, Via R. Cozzi 55, Milano 20125, Italy; 3 Paul-Drude-Institut für Festkörperelektronik, Leibniz-Institut im Forschungsverbund Berlin e.V., Hausvogteiplatz 5-7, Berlin 10117, Germany; 4 Institute for Microelectronics and Microsystems-IMM, Consiglio Nazionale delle Ricerche-CNR, Via del Fosso del Cavaliere 100, Roma 00133, Italy; 5 Department of Physics, Sapienza University of Rome, P. le Aldo Moro 2, Roma 00185, Italy; 6 Department of Chemical Science and Technologies, 9318 University of Rome Tor Vergata, Via della Ricerca Scientifica 1, Roma 00133, Italy

**Keywords:** GeTe-rich Ge-Sb-Te, vacancy ordering, DFT, valence band, molecular beam epitaxy, vdW epitaxy, XPS, phase-change materials

## Abstract

In this work, a study of the structural and electronic properties of epitaxial GeTe-rich (GeTe)_*m*_(Sb_2_Te_3_)_*n*_ alloys grown on Si substrate by molecular beam epitaxy is presented, with particular focus on the effects of annealing at increasing temperatures. The samples, displaying a lamellar structure stabilized by epitaxy, were investigated by X-ray diffraction and X-ray photoemission spectroscopy after heating in ultra-high vacuum. The combined use of these techniques, supported by density functional theory calculations, reveals compositional and structural changes induced by annealing, clarifying how the rearrangement of residual defects influences the stacking order of the epilayers. These results provide key insights into the vacancy ordering of GeTe-rich (GeTe)_*m*_(Sb_2_Te_3_)_*n*_, which are particularly relevant not only for memory applications but also in light of the recent discovery of (GeTe)_*m*_(Sb_2_Te_3_)_*n*_ ferroelectricity.

## Graphical abstract

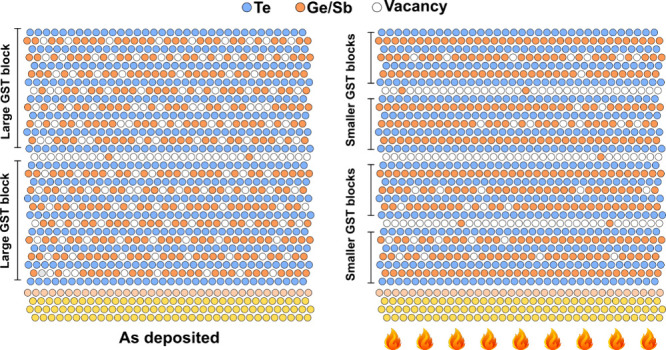

## Introduction

Chalcogenide phase-change materials (PCM) and, in particular, Ge–Sb–Te compounds (GST), are well-known materials exploited for the realization of electrical and optical non-volatile phase-change memory devices.^1^ The storage mechanism relies on the strong electrical/optical contrast between the amorphous and crystalline phase and on the possibility to reversibly switch between these two states upon application of an electrical/optical input of proper intensity and duration. The (GeTe)_*m*_(Sb_2_Te_3_)_*n*_ alloys along the related pseudobinary tie line were found to exhibit particularly interesting properties for memory devices, fulfilling important constraints for practical utilization such as high switching speed and large programming window. Indeed, it has been demonstrated that GST can switch between amorphous and crystalline phases on the nanosecond timescale.^2,3^ Interestingly, the compositional/structural properties of the material can strongly affect the alloy resistivity range: quasi-single crystalline GST were found to yield an increase of the resistivity up to 1 order of magnitude with respect to polycrystalline GST, while the ordering degree of vacancies is correlated to the metal–insulator transition observed in these alloys.^4^

Crystalline GST alloys (x-GST) display two different phases: a stable trigonal one (t-GST) and a metastable cubic rocksalt phase (c-GST), which is the one generally formed during the electrical switching in a memory device. Depending on the composition, x-GST contains a large number of stoichiometric vacancies in the cationic sublattice.^5^ The two x-GST phases differ in how these vacancies are arranged within the lattice. In c-GST, vacancies can be randomly distributed in the Ge/Sb sublattice (cubic-disordered phase) or partially ordered into not completely depleted vacancy layers (VLs) perpendicular to the [111] direction (cubic-ordered phase). Similarly, in t-GST vacancies are ordered to form van der Waals (vdW)-like gaps in between consecutive Te planes. Notably, the Te–Te distance across the gap is not considered to be a pure van der Waals (vdW) gap^6,7^ as it does not match the wider interlayer separations typical of 2D materials like graphene or transition metal dichalcogenides. Thus, the structure of these materials is more accurately described as a vdW-like stacking. As a result, both t-GST and ordered c-GST exhibit a lamellar structure, where each block consists of alternating Sb/Ge and Te layers, separated by either a vdW-like gap or a VL, respectively.^4,8^ The number of atomic layers composing each block (i.e., the block thickness), for both cubic-ordered and trigonal phases, depends on their composition (e.g.: 7 layers for Ge_1_Sb_2_Te_4_, 9 for Ge_2_Sb_2_Te_5_, 11 for Ge_3_Sb_2_Te_6_, and so on^4,9^ ).

In recent years, it has been shown that the use of textured periodic stacks of two or more PCM materials, usually Sb_2_Te_3_, and GeTe or GST, as active materials in memory devices leads to reduced programming currents and increased endurance.^10,11^ The origin of these improved performances has been much debated in the literature, but there is now some agreement that the layered nature of the SLs and the presence of vdW gaps within the films play a fundamental role.^10–15^ In particular, vdW gaps lower the cross-plane electrical and thermal conductivity, resulting in nanoscaled electro-thermal confinement that reduces the energy required for the amorphous-to-crystalline transition.^11^ A similar behavior has been observed in PCM devices based on textured GST alloys.^13,16^ Furthermore, the capability to epitaxially grow these materials has the potential to single out new physical properties. For example, the emergence of ferroelectric properties in epitaxial GeTe-rich (GeTe)_*m*_(Sb_2_Te_3_)_*n*_ (epi-GST) alloys has been recently reported.^17^ Such a material, fabricated by molecular beam epitaxy (MBE), has been shown to retain the layered structure typical of epitaxial GST, while hosting very thick blocks (e.g., 15 atomic layers or more), responsible for the ferroelectric behavior of the films. Indeed, despite the very high crystalline quality achieved by MBE, the films still feature a composition-dependent distribution of block sizes, known as compositional disorder.^17^ Therefore, the ability to control the texture of the films, the ordering of vacancies, the density of the vdW-like gaps as well as the composition of the blocks between the gaps, is of paramount importance.^15^ Moreover, given the direct impact of both vacancies and texturing on the metal-insulation transition and programming window,^4^ their tailoring is key for the development of devices operating on multiple states, and thus for in-memory computing applications.^18^

Within this framework, here we present a study of the electronic and structural properties of epitaxial GST films. The work focuses on the effects of annealing at increasing temperatures on the structural ordering of x-GST films with different GeTe concentrations. The GST films were grown by MBE, which has been proven to be the ideal technique to attain oriented films with high crystal quality.^17,19–21^ A combination of X-ray Photoemission Spectroscopy (XPS) carried out *in situ* after the annealing treatments, and *ex situ* X-ray diffraction (XRD) was used to gain direct information on composition, phase, electronic and structural properties of the films. Density Functional Theory (DFT) calculations were carried out to obtain the electronic density of states (DOS) of GST alloys with increasing GeTe content, to be compared to the experimental valence bands measured by XPS. We show that thermal annealing induces significant vacancy rearrangements in the epitaxial films, leading to a reduction in GST block size, and that this effect is more pronounced in GeTe-rich samples. Additionally, XPS analysis revealed that the block size does not always match the actual GST composition and that the core-level electronic properties remain largely unaffected by changes in the stacking order, indicating a weak correlation between the structural and electronic properties.

## Results and Discussion

To investigate the vacancy ordering in GeTe-rich GST alloys, we grew three epitaxial GST films on Sb passivated Si(111)–(√3 × √3)­R30°: one reference Ge_2_Sb_2_Te_5_ sample (GST225) and two GeTe-rich GST films with nominal compositions Ge_8_Sb_2_Te_11_ (GGST1) and Ge_15_Sb_2_Te_18_ (GGST2). Figure 1 shows the XRD symmetric ω*–2*θ profiles measured around the equivalent cubic (222) reflection for the GST225 (red squares), GGST1 (green circles), and GGST2 (blue triangles) samples, both as-grown (open symbols) and after annealing in ultra-high vacuum (UHV) and XPS characterization (closed symbols). The scans in Figure 1 show the typical pattern of epitaxial GST, with an intense (222) reflection (around 3.55–3.64 Å^–1^) due to the main Te–Te periodicity, and a broader and less intense structure at lower Q_*z*_ (VL peak) attributed to the VL periodicity along the [111] direction of the cubic cell.^4,19^ The lower Q_*z*_ position of the GST (222) reflection for the as-grown GGST1 and GGST2 samples testifies their higher GeTe content.^22^

**Figure 1:**
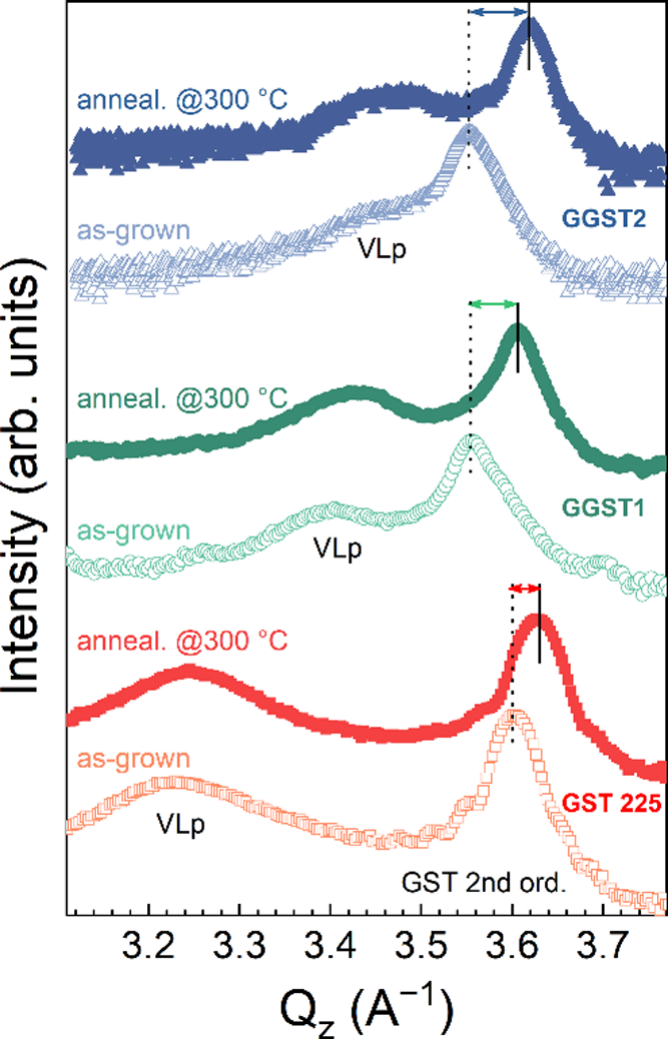
XRD profiles of GST225 (squares), GGST1 (circles) and GGST2 (triangles), acquired on the as-grown samples (open symbols) and after annealing in UHV at 300 °C (closed symbols). The shifts of GST(222) reflections, increasing the distance from the VLp, indicates a reduction in block size upon annealing.

From the Q_*z*_ separation δ_*Q*_*z*__ between the (222) reflection and the VL peak, the mean block size in the films could be estimated. As shown in Figure 1 for both the as-grown and the annealed samples, δ_*Q*_*z*__ (as evaluated by a fitting deconvolution of the XRD data) is much smaller in the GeTe-rich films than in the reference GST225, thus reflecting the increase of the mean block size when moving toward GeTe enriched phases.^17^ The average composition of the samples could then be estimated according to Da Silva et al.^9^ from the mean block size, obtaining the corresponding layers stacking along the [111] direction (7 atomic layers for GST 124, 9 for GST 225, 11 for GST 326 and so on). Results are summarized in Table 1. The broadening of the VL peaks denotes the presence of compositional disorder (i.e., the coexistence of GST blocks with different number of layers), typically affecting epitaxial GST alloys.^4^ As previously shown by Cecchi et al.,^17^ due to the reduced number of VLs per unit length in GeTe-rich GST films, the resulting lamellar structure is less ordered.

**Table 1:** Average Stacking Estimated via XRD of As-Grown and 300 °C Annealed GST Films along with Composition Evaluated via XPS after Annealing at 260 and 300 °C in UHV

	average stacking (XRD)	composition (XPS)		average stacking (XRD)
sample	as-grown	annealing @260 °C	annealing @300 °C	annealing @300 °C
**GST225**	GST 2 2 5	GST 1 2 4/2 2 5	GST 1 2 4/2 2 5	GST 1 2 4/2 2 5
**GGST1**	GST 8 2 11	GST 9 2 12	GST 7 2 10	GST 7 2 10
**GGST2**	GST 15 2 18	GST 5 2 8	GST 4 2 7	GST 9 2 12/10 2 13

The measurements in Figure 1 also show a clear increase of δ_*Q*_*z*__ after the annealing, in particular for the GGST samples. This behavior suggests that the thermal treatments induce structural changes leading to a reduction of the mean block size. Consequently, for all samples the average layer periodicity shrinks, corresponding to GST compositions less rich in GeTe (see Table 1). This effect is particularly evident for GGST2, which is the sample with, nominally, the highest as-grown GeTe content. Hence, assuming that the chemical composition in the films remains fairly unchanged, the present findings highlight that, in the as-grown films, a part of the vacancies is still disordered, i.e. the GST blocks formed during the epitaxial growth are thicker than expected, and only upon annealing they rearrange forming additional VLs.

The XRD characterization allowed the evaluation of the epitaxial quality of the as-grown and annealed films as a function of composition. First, all the annealed samples exhibit a shift of the GST (222) main peak toward higher Q_*z*_, indicating a reduction of the average Te–Te distance. Such a change is mainly given by the reduction in the number of atomic layers in the average GST block.^22^ Moreover, a smaller Te–Te distance may be compatible with two other thermally activated mechanisms: (i) the presence of residual domains with cubic stacking in the as-grown film^4^ which turn trigonal upon annealing, and (ii) the complete depletion of the VLs in the trigonal phase to form pure vdW-like gaps. In fact, according to Zhang et al.,^23^ the trigonal structure is energetically favorable also in the case of nonfully depleted VLs.

The change in full width at half maximum (FWHM) of both GST(222) and VL peaks upon annealing provides an interesting comparison between the samples and their reconfiguration. For GST225, the slight broadening of the main peak is ascribed to a reduction of the film thickness, likely driven by desorption in UHV during the annealing. At the same time, the FWHM of the VL peak decreases (−19%), indicating that the compositional disorder has been reduced. Interestingly, for the GGST samples we recorded an opposite trend. It is evident from Figure 1 that, in both cases, the as-grown GeTe-rich films show broader (222) reflections than GST 225, which become narrower after annealing. Such an effect clearly indicates an improvement of the crystal quality of the films. Meanwhile, the FWHM of the VL peak increases (+20% and +49% for samples GGST1 and GGST2, respectively), suggesting that, while thermal treatment promotes the ordering of residual vacancies into layers, it also increases the size distribution of the GST blocks.

The XRD results showed that highly oriented epitaxial GeTe-rich GST can be grown with very thick blocks intercalated by vdW gaps or partially depleted VLs. Also, our results revealed that the annealing process triggers a structural rearrangement leading to an improvement of the crystal quality and, particularly, to a reduction of the average block size (see Table 1). We tentatively attributed this structural rearrangement to the presence of an excess of vacancies within the GST blocks of the as-grown GGST samples (see Figure 1).

To get further insights into the effects of annealing and gain a direct indication of the composition evolution, a complete XPS characterization was performed. As described in the experimental and methods section, to prevent the films surface from oxidation and contamination, as-grown samples were capped with a layer of amorphous Te that was thermally removed in the XPS analysis chamber at T = 260 °C. In Figure 3 a stack of Te 4d, Sb 4d, Ge 3d shallow core levels for the GeTe-rich GST samples is shown: GGST1 and GGST2 (blue triangles and green circles, respectively) and the GST225 sample (red squares) collected after the decapping process at T = 260 °C and after a further annealing at 300 °C for 20 min. For all the samples, the removal of the Te cap was confirmed by the observation of a clear hexagonal low energy electron diffraction pattern (see the inset of Figure S1 of Supporting Information - SI), as expected for epitaxial GST oriented along the [111] direction of the cubic cell, and by the appearance in the XPS spectra of the Ge 3d, Sb 4d, and Ge 2p core levels at about 30, 33, and 1218 eV, respectively. In Figures S1 and S2 of SI, the complete XPS characterization of GGST2 is reported, including the Te 3d and Ge 2p core levels.

Quantitative information from XPS could be obtained after a fit deconvolution of the spectra of the Te 4d, Sb 4d and Ge 3d core levels (see also refs 24 and 25). For the best fitting of all the spectra, we considered one main contribution (one Voigt doublet) associated with each element of GST at 40.2, 32.8, and 29.7 eV, for Te 4d_5/2_, Sb 4d_5/2_ and Ge 3d_5/2_, respectively. The fit results are shown in Figure 3, where the total fit is superimposed as a black continuous line on the experimental data points and the individual Voigt doublets attributed to each core level are explicitly shown in purple (Te 4d), gray (Sb 4d) and pink (Ge 3d). Besides the contributions associated with the main GST phase, one and two Voigt doublets were added at the higher binding energy (BE) side for the fitting of the Te 4d and Ge 3d core levels, respectively. The former accounts for the presence of metallic Te, residual from the capping layer (it vanishes after the second annealing at 300 °C), and the latter for minimal germanium oxides and suboxides formed during the decapping procedure^26^ from the oxygen present on the Te-cap (see also Ge 2p core levels in Figure S2). The complete output of the best fit is reported in Table S2 in the SI. The results in Figure 3 pointed out that, when moving from GST225 to the GGST samples, the spectra of Te 4d, Sb 4d and Ge 3d core levels do not show changes in line-shape or chemical shifts of the main components (see fitting results in Table S2), but only their relative intensities are affected. Such modulation arises from a change in composition from sample to sample and/or upon annealing (see below and Table 1).

The results of the XPS compositional analysis conducted after each annealing step, along with the XRD stacking data, are reported in Table 1. It is important to note that, while XPS provides the actual composition of the films, XRD estimates the average size of the GST blocks from which one can infer the composition. However, due to the presence of vacancies, this composition may differ from the actual atomic composition. This effect is particularly evident in GeTe-rich samples: while the XPS-derived composition and XRD-derived stacking are closely comparable for GST225, clear variations are observed in GGST as a function of annealing. For instance, in GGST2, which is the GeTe-richest film, a significant discrepancy is found between the as-grown XRD stacking and the XPS composition after the first annealing at 260 °C. Furthermore, Table 1 shows that after annealing at 300 °C, while the compositions of GST225 and GGST1 determined by XPS match the ones determined by XRD, a notable discrepancy remains in GGST2. These findings indicate that, while MBE enables the growth of GST with the desired stacking for a given GeTe-rich composition, the films can contain a substantial number of excess vacancies. These vacancies contribute to the formation of large blocks whose composition may significantly differ from what is predicted on the basis of the block size. Moreover, thermal annealing triggers the rearrangement of the vacancies, leading to a more energetically favorable stacking that ideally aligns with the actual atomic composition, as observed in GST225 and GGST1. Nevertheless, in the case of larger blocks characterized by a higher concentration of vacancies (e.g., GGST2), this reorganization may require more energy and, consequently, longer annealing. This highlights the crucial role of kinetics in the ordering process. We speculate that the reorganization primarily occurs within the GST blocks themselves, for example from stacking faults of incomplete VL, rather than across block boundaries (see Figure 2). This behavior is consistent with the findings of ref.,^27^ which demonstrated that well-defined vdW-like gaps hinder cation diffusion across interfaces, thereby supporting the idea that vacancy redistribution in our films is predominantly governed by intrablock diffusion dynamics. It is important to note that, although small, XPS shows minimal changes in compositions after annealing likely due to preferential diffusion of Sb and Te.^28^ The fit deconvolution of the spectra also shows a reduction of the FWHM of the core levels after annealing, in particular for GGST samples (see Table S2). This behavior confirms an improvement of the crystal quality of GeTe-rich films after annealing, as suggested by XRD.

**Figure 2:**
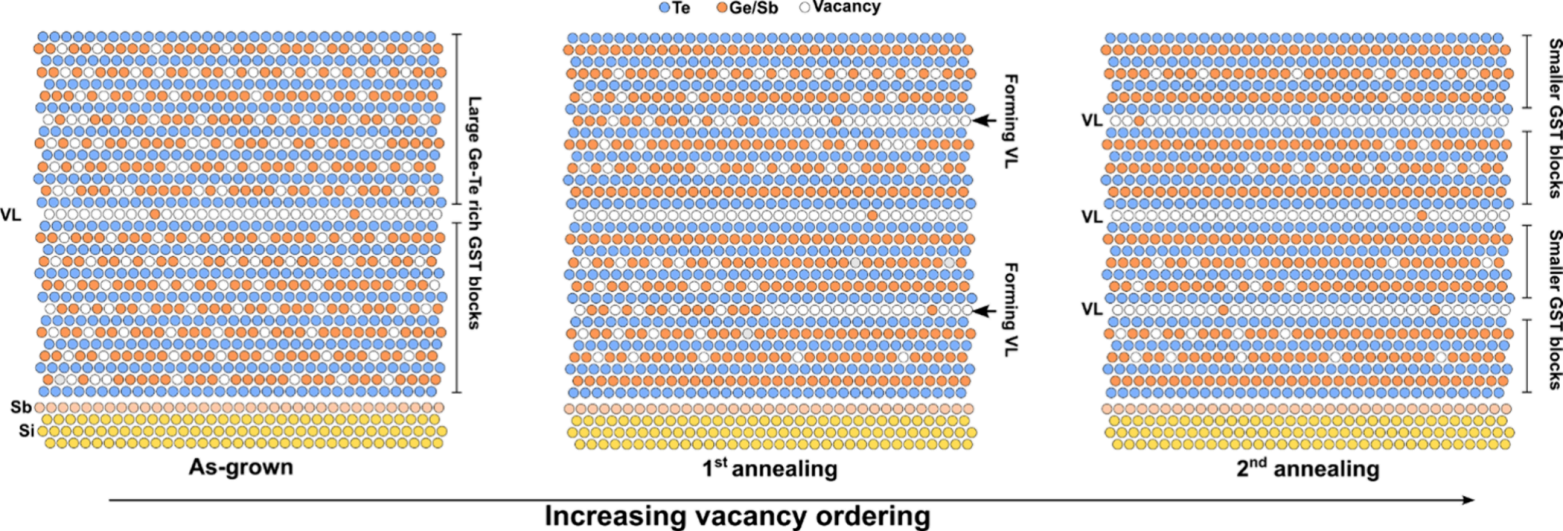
Schematics of the vacancy ordering process upon annealing. Te atoms are represented by light blue circles, Ge and Sb by orange ones, vacancies by empty circles. Starting from an as-grown GST characterized by large blocks with excess vacancies, thermal annealing induces a gradual redistribution that leads to the formation of new vacancy layers and, ultimately, of smaller blocks.

In summary, XPS analysis suggested that the electronic properties of the epitaxial GST investigated here are not as strongly affected by composition variations as the structural properties: while the XPS spectra in Figure 3 are essentially comparable (regardless of the intensities), the XRD scans of GST samples reported in Figure 1 show changes after annealing in the position and/or intensity and shape of both the GST (222) reflection and the VL peak.

**Figure 3:**
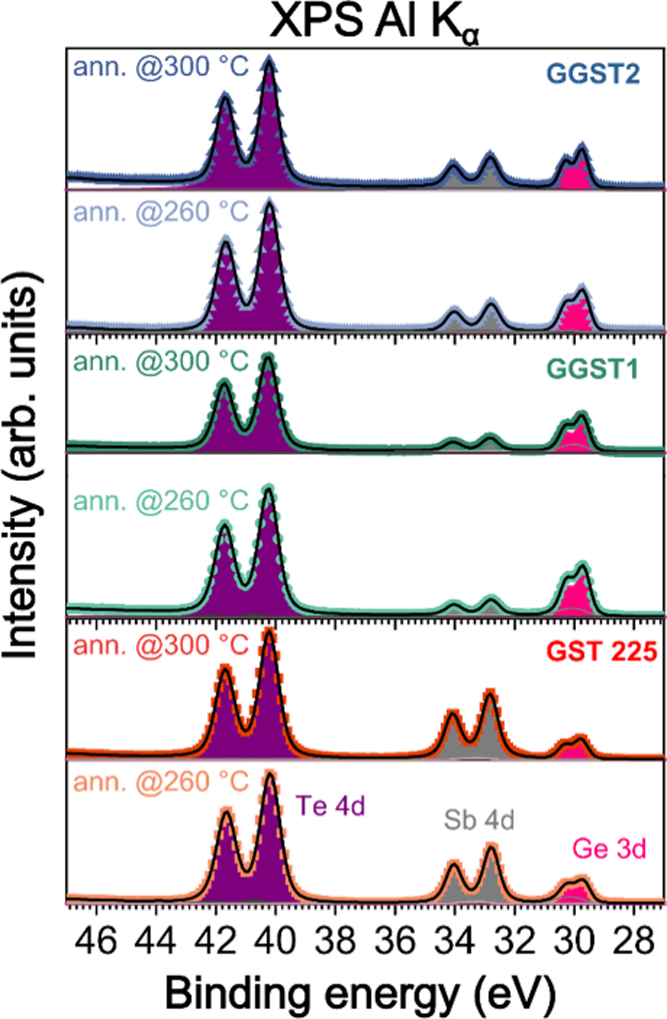
From bottom to top: XPS spectra of the shallow Te 4d (purple), Sb 4d (gray) and Ge 3d (pink) core levels for GST225, GGST1 and GGST2 (red squares, green circles and blue triangles, respectively), after thermal removal of the Te cap at 260 °C and after a successive annealing at 300 °C. The results of fitting deconvolution are shown as black continuous lines. The quantitative core levels analysis reveals the evolution of sample composition upon annealing.

In fact, XPS results show that, for different stacking (i.e., average number of atomic layers in the blocks), the Ge, Sb and Te core levels do not change significantly. This occurs regardless of how the change of composition is obtained, whether through annealing or by growing films with different nominal composition. Precisely, for the GST225, GGST1 and GGST2 samples, we did not observe any chemical shift (Figure 3) of the core levels of Ge and Sb (at least within the energy resolution of our XPS setup), which could occur in the presence of inequivalent lattice sites for Ge and Sb atoms. While this is not the case for the cubic phase, it could be possible for the trigonal one. Actually, for t-GST along the pseudobinary line, two different configurations are most favorable:^29^ the Kooi-like, where Sb atoms are in the outermost layers of the block (close to the VLp/vdW gaps), and the disordered one, with Sb and Ge randomly occupying the cationic layers. In the case of GGST with a very high GeTe content and a Kooi stacking, the Ge 3d core levels from atoms in the middle of the blocks could shift toward higher BE as expected for pure GeTe.^26^ However, while the XRD data confirm that the average block size of GST increases with GeTe content (Figure 1, Table 1), the XPS spectra show no trace of chemical shifts or secondary components. On the one hand, this could suggest a disordered configuration. On the other, the reduction in average block size after annealing greatly lowers the differences between potential inequivalent sites. Therefore, no definitive conclusions could be drawn from these results.

Further information on the electronic properties of the samples could be obtained from the VB measurements performed by XPS. The results are summarized in Figure 4(a), where the VB spectra of the samples after annealing at 260 and 300 °C are shown. In all spectra, the typical line-shape of crystalline GST^24,25,30^ can be identified with two distinct regions: the one located between 14 and 6 eV, accounting for the contribution of the Te 5*s* levels (peak labeled as A at about 12 eV), the Sb 5s and Ge 4s levels (peak B, between 11 and 7 eV), and the VB region at lowest BEs (0–7 eV, labeled as C). The broad VB band up to the Fermi level has main p-type character due to the contribution of Ge 4p, Sb 5p and Te 5p states, with Ge 4p at higher energy and Te 5p at the lowest.^24,25,30–32^ For each annealing series, the peak B shows an evident change in shape, intensity and position, moving from GST225 to GGST samples, reflecting the increased contribution from Ge 4s states in GeTe-rich compositions (see Table 1). In region C, we identified three clear features for GST225 (bottom curves in Figure 4(a)) at about 1 eV (C_1_), 2 eV (C_2_), and 3.2 eV (C_3_) with a shoulder around 4 eV. The energy region of the C_2_ and C_3_ peaks is where the major contribution from Ge p states is expected.^31,32^ Actually, in the case of GeTe-rich samples (middle and top curves in Figure 4 (a)), the peaks C_2_ and C_3_ slightly shift toward higher and lower BEs, respectively, resulting less defined and partly convoluted; at the same time, C_1_, which has a major p-type contribution from Te, becomes more evident.^31,32^ A shoulder arises at about 5.5 eV, particularly evident for sample GGST1.

**Figure 4:**
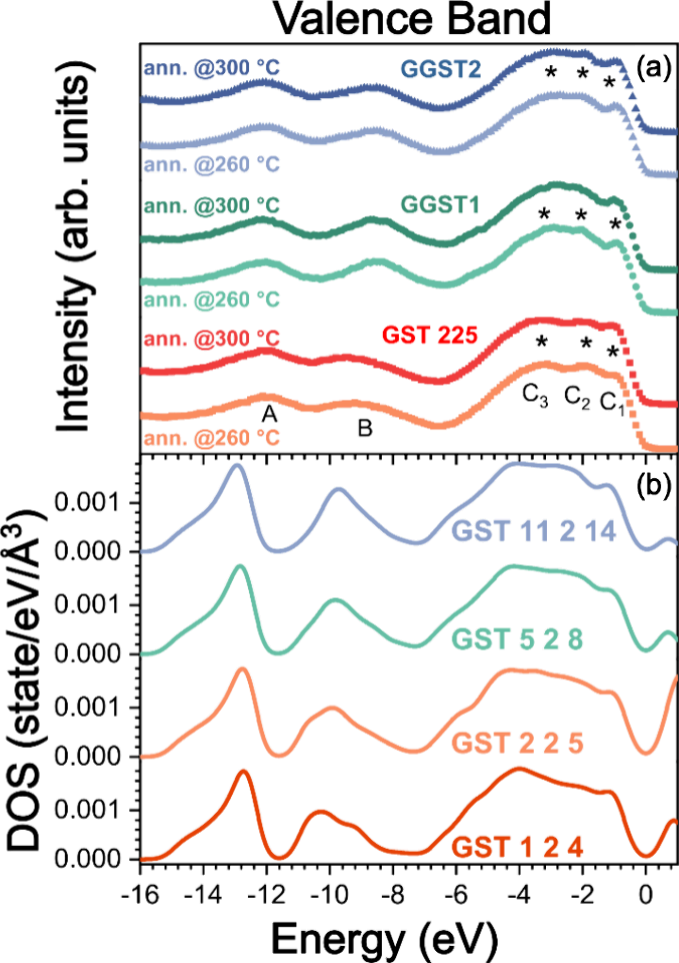
(a) Experimental VB measured after annealing in UHV at 260 and 300 °C for GST225 (red squares), GGST1 (green circles) and GGST2 (blue triangles). (b) Theoretical DOS of the trigonal phase of pseudobinary GeTe-Sb_2_Te_3_ compounds of different compositions, computed by DFT with the HSE06 hybrid functional. The close correspondence between experimental and theoretical results allows to clearly identify the contribution of each atomic species to the VB structure. The highest occupied Kohn–Sham (KS) state was taken as reference (energy zero).

The experimental VB spectra show very close correspondence with the theoretical DOS computed by DFT and plotted in Figure 4(b) and the projected DOS in Figure S3 of the Supporting Information, where t-GST is modeled with a Kooi stacking, recently employed in modeling the emergence of ferroelectric distortion in GeTe-rich epitaxial GST.^17^ In particular, the noticeable contribution in the DOS from Te, Sb and Ge s-states in between 7 and 15 eV of BE and the increase around 9.5 eV due to Ge 4s-states (see projected DOS in Figure S3) can be clearly observed as the GeTe content increases. In the BE region 0–7 eV, the theoretical DOS for GST124 shows three features at about 1, 2, and 4 eV, as well as a weak shoulder at about 5.5 eV. These three features correspond to the C1, C2 and C3 peaks of the experimental spectra. The DOS projected on atomic orbitals (see Figure S3 in the Supporting Information) confirms that Ge 4p mainly contributes to the peak C2 while Sb 5p mainly contributes to the peak C3. The contribution of Ge and Sb is instead much lower in the peak C1. We remark, however, that Te 5p is the main contribution to all C1, C2 and C3 peaks (see Figure S3). With increasing GeTe content, the peak at about 4 eV in the DOS appears slightly more structured, while the shoulder at 5.5 eV is much more evident and shifted at about 6 eV. The features singled out in the 0–6 eV region agree with peaks C_1_, C_2_ and C_3_ and the shoulders on the higher BE side of the experimental spectra for GST225 and GGST samples, although the increased contribution from Ge 4p states in GGST samples makes the C_2_ and C_3_ features less resolved.

Hence, our analysis suggests that the VB does not undergo substantial changes at increasing GeTe content, if the intensity variation of some features and the partial convolution of others are excluded. This finding is perfectly in line with the observed evolution of the shallow core levels reported in Figure 2, confirming that the electronic properties are quite insensitive to the stacking ordering.

Figure 5 shows a close-up of the BE region around the valence band maximum (VBM), where a general trend can be observed for all the samples: after annealing at 300 °C, the VBM position always shifts toward higher BEs. Furthermore, the position of the VBM of the GST225 samples is always found at higher BEs than in GGST samples. These shifts can be interpreted considering that crystalline GST typically displays a large concentration of nonstoichiometric vacancies that shift the Fermi level inside the valence band, accounting for the p-type metallic conduction of GST.

**Figure 5:**
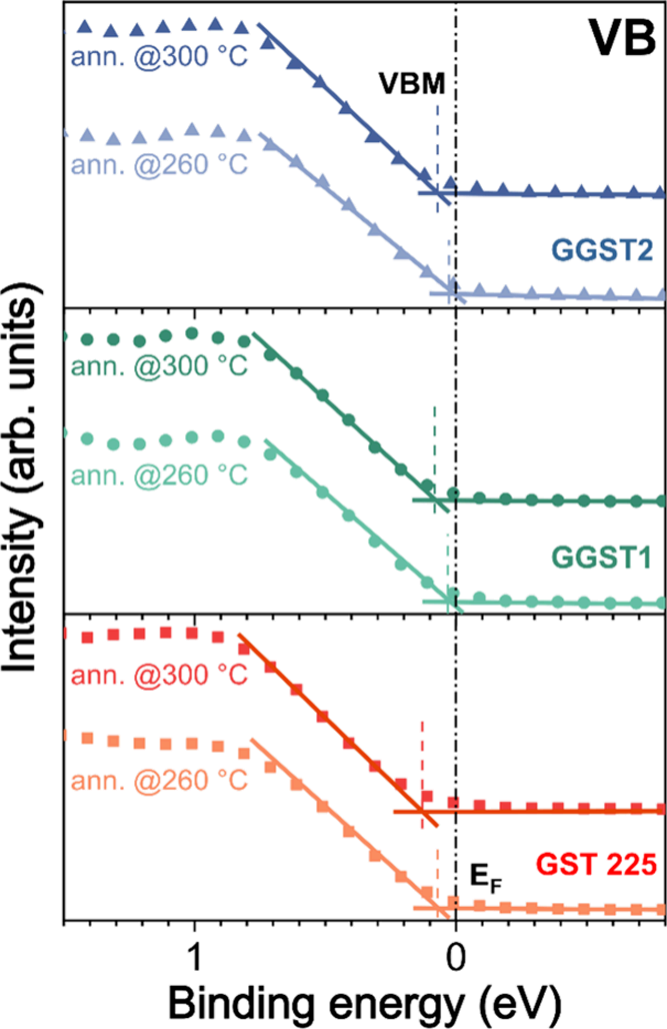
VBM for GST225, GGST1 and GGST2 after in situ annealing at 260 and 300 °C. VBMs exhibit a systematic shift toward higher BEs linked to the reduction of vacancies induced by thermal annealing.

The GGST samples exhibit a VBM closer to the Fermi level than that of GST225, which means a more metallic character, thus suggesting the presence of an excess of vacancies. This result corroborates our hypothesis that the epitaxial growth of GeTe-rich samples can stabilize the presence of an excess of vacancies within the thicker GST blocks. On the other hand, after annealing the GGST samples, we observed a shift of the VBM toward higher BEs. This behavior is compatible with a structural rearrangement involving a vacancy ordering, a reduction of their concentration^32,33^ and the formation of GST blocks of smaller size.

## Conclusions

This study presents a structural and electronic characterization of epitaxial GeTe-rich GST films grown by MBE. The results obtained from XRD and XPS measurements pointed out that lamellar GST with GeTe enriched composition of high crystal quality can be stabilized by epitaxy. Furthermore, the combined use of XPS after *in situ* annealing and *ex situ* XRD allowed us to study the compositional and structural changes induced by the thermal treatment, revealing evidence of structural rearrangements affecting the stacking ordering of the GST blocks. In general, we observed that, although XRD is the most suitable technique for assessing GST stacking, the XPS data show that the average block size does not necessarily match the actual GST composition. Notably, in GeTe-rich samples, a substantial difference between block size and composition arises due to the presence of excess vacancies within the lattice. Upon annealing, these vacancies reorganize into layers, leading to the formation of smaller GST blocks and reducing the discrepancy between block size and the effective composition. However, in nominally GeTe-richer films with as-grown larger block sizes (i.e., higher excess vacancy concentrations), this discrepancy may persist, depending on the annealing temperature and duration. This behavior accounts for the observed shifts of the VBM, which moves to higher BE as a consequence of the progressive vacancy ordering. Such results shed new light on the vacancy ordering of GeTe-rich GST, which are highly significant not only for phase-change memory applications, but also in the context of the recent breakthrough in GST ferroelectricity.

## Experimental and Methods

### Sample Preparation and Characterization

The epitaxial GST films, ≈20 nm-thick, were grown by MBE on Si(111)–(√3 × √3)­R30°–Sb passivated substrates.^34^ A ≈15 nm-thick capping layer of amorphous Te was deposited on top of the GST films to prevent the sample surface from oxidation during transfer in ambient atmosphere from the growth chamber to the analysis chamber. The samples were fabricated by changing the GeTe content in the film to achieve GeTe-rich GST compositions lying along the pseudobinary Sb_2_Te_3_–GeTe tie line. In particular, a reference Ge_2_Sb_2_Te_5_ sample (GST225)^35^ and two GeTe-rich GST films with compositions Ge_8_Sb_2_Te_11_ (GGST1) and Ge_15_Sb_2_Te_18_ (GGST2) were obtained by varying the fluxes of the constitutive elements during the growth.^17^

After deposition, the samples were extracted from the growth chamber and transferred to the analysis chamber, where they were characterized by XPS. The XPS measurements were performed by using a monochromatized Al Kα (1486.6 eV) X-ray source, a SPECS PHOIBOS 150 analyzer and 2D CMOS detector. A mild Ar+ sputtering was performed to remove the TeO_*x*_ (approximately 2–5 nm) formed during the transfer in air from the top layers of the sample surface and prevent oxidation of the underlying GGST surface.^26^ Next, the sample was annealed at first for 20 min at 260 °C in UHV, and then again at 300 °C for the same time. Different core levels were studied including Te 3d, Sb 3d, Te 4d, Sb 4d, Ge 3d, Ge 2p and the valence bands (VBs) also before thermal desorption of the Te cap and before and after each annealing. The collected XPS spectra were analyzed and fitted by the KolXPD software (http://kolxpd.com) using doublet Voigt functions and Shirley background. The composition of the films was determined from the area of the synthetic peaks used in the fitting process, following the equation:

$$x_{i}=\frac{\frac{A_{i}}{S_{i}}}{\underset{i}{\sum }\frac{A_{i}}{S_{i}}}$$ 1

where *A*_*i*_ represents the area of the Voigt curve fitting the core level of the *i*-th element in the film, *S*_*i*_ is its sensitivity factor, and *x*_*i*_ denotes its atomic percentage. The XRD measurements were performed on the as-grown samples and after the XPS experiments (annealed samples). The XRD characterization was carried out in the symmetric ω*-2*θ geometry using a Bruker D8 Discover diffractometer, equipped with a monochromatized Cu X-ray source employing the Kα_1_ X-ray radiation. The data analysis was carried out using *X-rayutilities* ^36^

## Density Functional Theory Simulations

The electronic DOS of Ge-rich GST pseudobinary compounds were computed within the DFT framework. We modeled the hexagonal phase of Ge_1_Sb_2_Te_4_ (GST 124), Ge_2_Sb_2_Te_5_ (GST 225), Ge_5_Sb_2_Te_8_ (GST 528) and Ge_11_Sb_2_Te_14_ (GST 11 2 14) with primitive cells containing 7, 9, 15, and 27 atoms, respectively. The Kooi stacking was considered for GST 225 and a Kooi-like stacking was chosen for GST 528 and GST 11 2 14 (i.e., Sb atoms are in the outermost cationic layers, close to the vdW gaps). For GST 11 2 14 we considered the model studied in ref.,^17^ which has been shown to display ferroelectric order in the inner GeTe-like part of the slab.

All the models were optimized (atomic positions and lattice parameters) with the Perdew–Burke–Ernzerhof (PBE) exchange and correlation functional,^37^ supplemented by a semiempirical correction due to Grimme (D2),^38^ norm conserving pseudopotentials and a plane wave expansion of the KS orbitals up to a kinetic cutoff of 35 Ry, as implemented in the Quantum-Espresso suite of programs.^39^ The integration of the Brillouin Zone (BZ) was performed over a 12 × 12 × 12 (GST 124), 12 × 12 × 6 (GST 225), 12 × 12 × 4 (GST 528) and 12 × 12 × 2 (GST 11 2 14) meshes. The optimized equilibrium lattice parameters *a* and *c* in the hexagonal setting are reported for all models in Table S1 in the Supporting Information. The DOS of the optimized models at zero temperature was computed from KS orbitals by employing the HSE06 hybrid functional,^40^ which better reproduces the band gap and the width of the electronic bands. The KS states were broadened by a Gaussian function with a variance of 0.03 Ry.

## Supplementary Material



## Notes

Description of the complete XPS characterization of GGST and fitting parameters; Additional details on the modeling of pseudobinary GeTe-Sb_2_Te_3_ compounds of different compositions in the hexagonal setting (PDF)
