# Supplementary material for: Electronic Properties and Stacking Ordering in Layered GeTe-Rich (GeTe) m (Sb2Te3) n
Source: ACS Appl Electron Mater. 2025 Oct 7;7(20):9320–8. doi: 10.1021/acsaelm.5c01185 (PMC12573760; doi:10.1021/acsaelm.5c01185)
Supplement: Supplementary file 1 [file el5c01185_si_001.pdf]

Electronic properties and stacking ordering in layered GeTe-rich $(GeTe)_m(Sb_2Te_3)_n$

Flavia Righi Riva[1], Stefano Cecchi[2,3*], Simone Prili[1,4*], Omar Abou El Kheir[2], Ernesto Placidi[5], Marco Sbroscia[5], Adriano Diaz Fattorini[1,4], Sabrina Calvi[1,4], Massimo Longo[6], Marco Bernasconi[2], Raffaella Calarco[4] and Fabrizio Arciprete[1,4]

[1] Department of Physics, University of Rome Tor Vergata, Via della Ricerca Scientifica 1, 00133 Roma, Italy

[2] Department of Materials Science, University of Milano-Bicocca, Via R. Cozzi 55, 20125 Milano, Italy

[3] Paul-Drude-Institut für Festkörperelektronik, Leibniz-Institut im Forschungsverbund Berlin e.V., Hausvogteiplatz 5-7, 10117 Berlin, Germany

[4] Institute for Microelectronics and Microsystems-IMM, Consiglio Nazionale delle Ricerche-CNR, Via del Fosso del Cavaliere 100, 00133 Roma, Italy

[5] Department of Physics, Sapienza University of Rome, P. le Aldo Moro 2, 00185 Roma, Italy

[6] Department of Chemical Science and Technologies, University of Rome Tor Vergata, Via della Ricerca Scientifica 1, 00133 Roma, Italy

* stefano.cecchi@unimib.it, simone.prili@roma2.infn.it

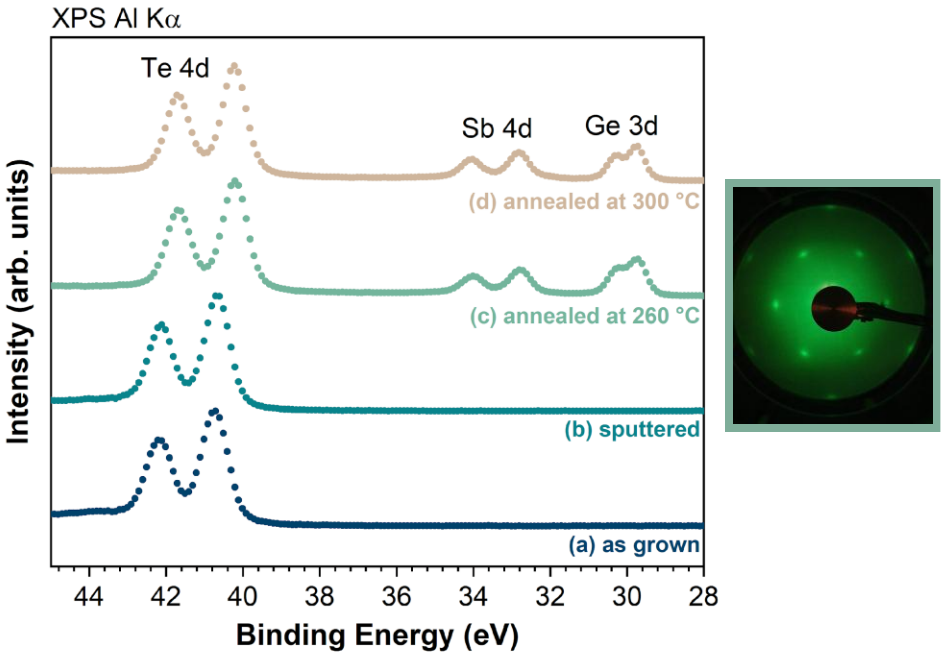

Figure S1: XPS spectra of the shallow Te 4d, Sb 4d and Ge 3d core levels of GGST2 sample: as-grown (a), after mild Ar^+ sputtering (b), after annealing at 260 °C (c), and after annealing at 300 °C (d). Contrary to (a) and (b), the spectra reported in (c) and (d) show the presence of Sb 4d and Ge 3d core levels originating from the GST

phase. The Te 4d doublet in (a) and (b) is found at the typical BE value of metallic Te (40.7 eV) and shifts toward lower BE, to the BE of GST, after heating of the sample. In the inset, LEED pattern of GGST1 after Te-decapping.

Figure S1 shows the XPS spectra of the Te 4d, Sb 4d, Ge 3d shallow core levels collected on the GeTe-rich film GGST2 (a) as-grown and after (b) Ar^+ sputtering and annealing at (c) 260 °C and (d) 300 °C. Contrary to (a) and (b), the spectra of the sample after annealing both display the Ge 3d (30 eV), and Sb 4d (33 eV) core levels doublets originated from the GST layer. By inspection of Figure S1, a shift of the Te 4d core levels can be detected in (c), with Te $4d_{5/2}$ moving after annealing from 40.7 eV of metallic Te (sample as-grown and sputtered) to 40.2 eV of Te in GST lattice. An analogous trend can be observed for the Te 3d levels (see Figure S2). Since the BE of the Te 4d and Te 3d levels in both the as grown and the sputtered samples is compatible with the one of metallic Te, we conclude that the complete desorption of the Te capping layer is not induced by a mild Ar^+ sputtering but requires a subsequent heating of the samples. This is confirmed by all the above observations concerning the appearance in (c) and (d) of the Sb and Ge core levels peaks previously listed and attributed to the GST phase (see Figure S1, and Figure S2). Furthermore, the XPS spectra of the Te 3d levels of the as-grown sample and after Ar^+ sputtering reported in Figure S2 (spectra (a) and (b) respectively) only reveal small differences but a drastic reduction in the intensity of the peak at 576 eV associated to TeO_x compounds. These observations confirm that the conditions employed during Ar^+ sputtering can successfully promote the almost complete removal of superficial TeO_x formed upon oxidation of the Te capping layer following samples exposure to air. Minimal amounts of oxygen are still detectable in the form of GeO_x compounds (see Figure S2 (c) and (d)).

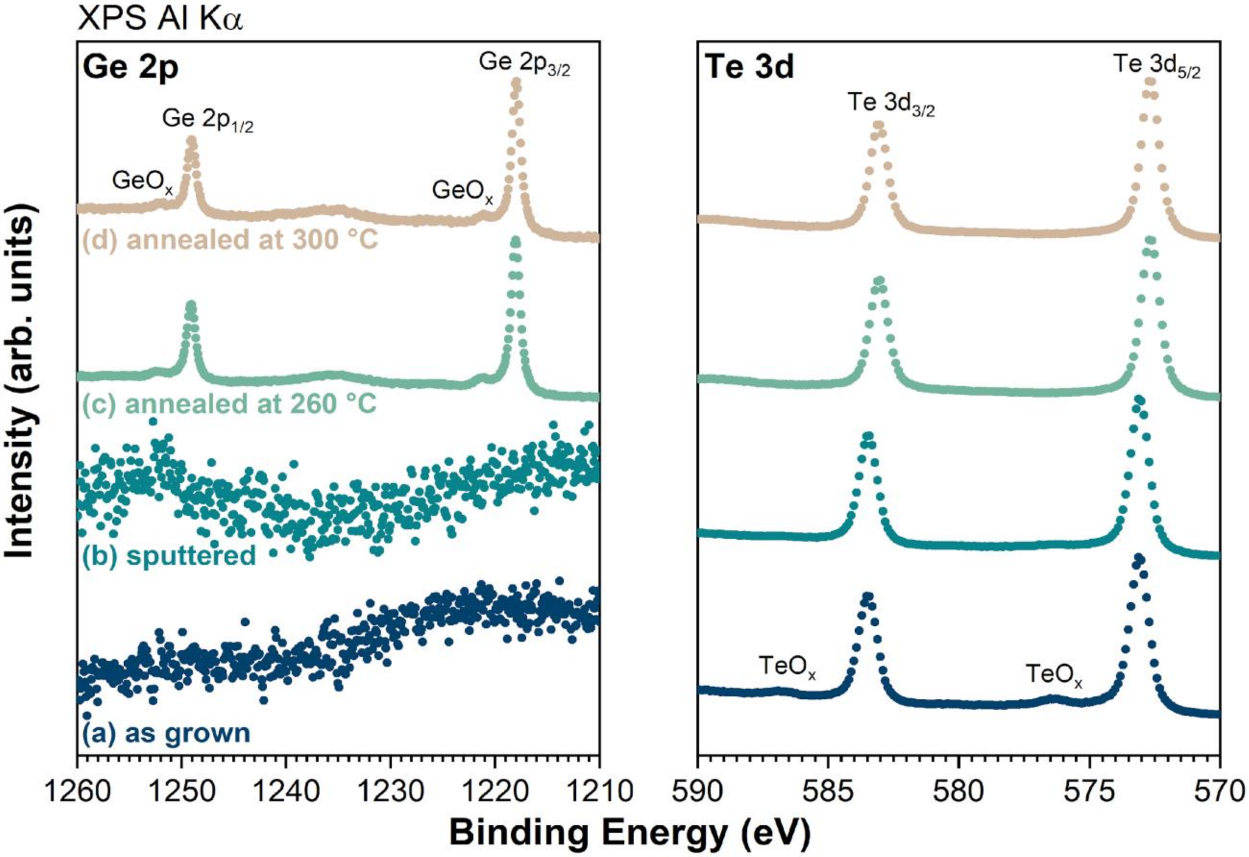

Figure S2. XPS spectra of the Ge 2p (left panel) and Te 3d (right panel) core levels measured on the GeTe-rich sample GGST2 (a) as-grown and after (b) mild Ar+ sputtering followed by (c) annealing at 260 °C and (d) annealing at 300 °C. As opposed to (a) and (b), the spectra (c) and (d) reported in the left panel clearly reveal the presence of Ge 2p core levels originating from the GST layer. As already observed for Te 4d shallow core levels (see Figure S1), the position of the Te 3d levels shifts from the characteristic value of metallic Te (about 575 eV) toward lower BE upon sample heating at 260 °C (spectra (c) and (d) in the right panel). The Te 3d levels collected on the sample as-grown and after Ar+ sputtering (spectra (a) and (b) in the right panel, respectively) also reveal a drastic reduction after sputtering in the intensity of the peaks associated to TeO_x compounds formed upon surface oxidation of the Te capping layer after sample exposure to air.

Table S1. Optimized lattice parameters at the DFT-PBE level of the GST models in the hexagonal setting.

**Lattice parameters**	GST 124	GST 225	GST 528	GST 11 2 14
**a (Å)**	4.210	4.191	4.174	4.202
**c (Å)**	13.630	17.062	27.188	47.445

Table S2. Fitting parameters for Te $4d_{5/2}$, Sb $4d_{5/2}$ and Ge $3d_{5/2}$ core levels.

**Sample**	**Level**	**Annealing at 260 °C**		**Annealing at 300 °C**	
		**Position (eV)**	**FWHM (eV)**	**Position (eV)**	**FWHM (eV)**
GST225	Te $4d_{5/2}$	40.2	1.76	40.2	1.71
	Sb $4d_{5/2}$	32.8	1.41	32.8	1.42
	Ge $3d_{5/2}$	29.7	1.20	29.8	1.23
GGST1	Te $4d_{5/2}$	40.2	1.73	40.2	1.68
	Sb $4d_{5/2}$	32.7	1.43	32.8	1.43
	Ge $3d_{5/2}$	29.7	1.17	29.7	1.13
GGST2	Te $4d_{5/2}$	40.2	1.72	40.2	1.65
	Sb $4d_{5/2}$	32.8	1.42	32.8	1.39
	Ge $3d_{5/2}$	29.7	1.22	29.7	1.14

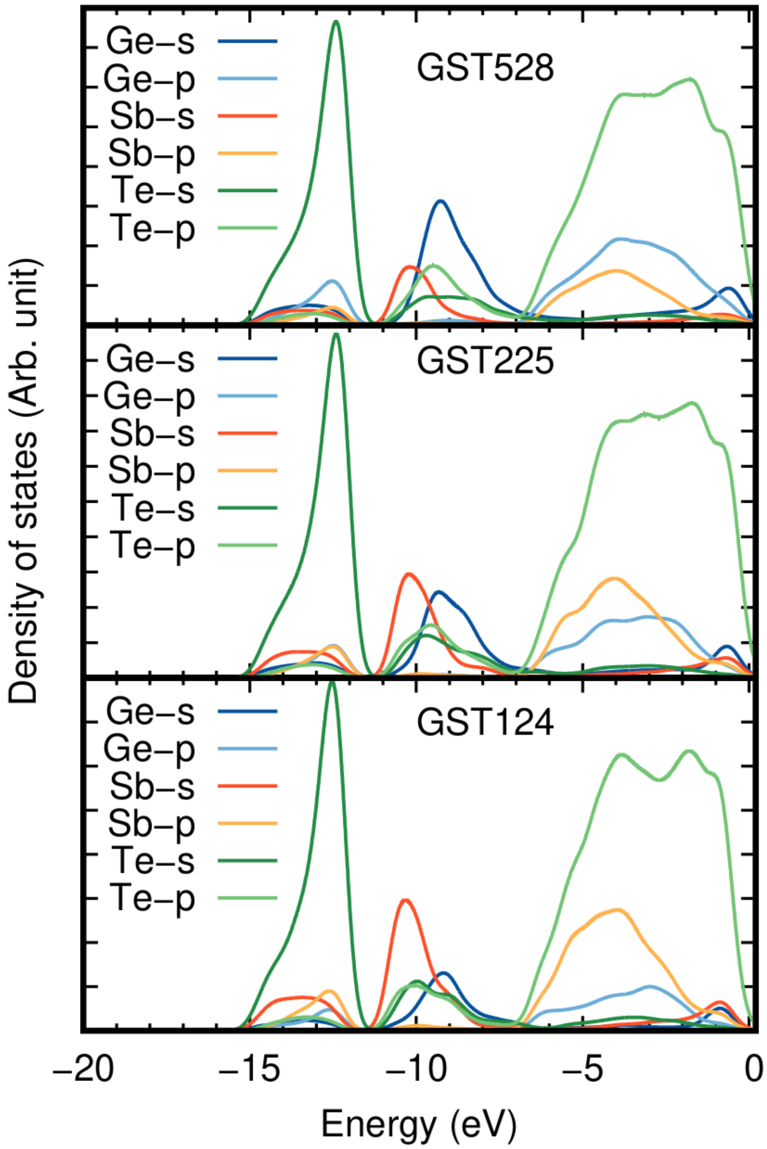

Figure S3: Electronic density of states of the trigonal GST124, GST225, GST528 projected on atomic s and p pseudowavefunctions.
